# Tuberculous meningoencephalitis associated with brain tuberculomas during pregnancy: a case report

**DOI:** 10.1186/s13256-017-1347-7

**Published:** 2017-06-29

**Authors:** Sadie Namani, Shemsedin Dreshaj, Arieta Zogaj Berisha

**Affiliations:** 1 Clinic of Infectious Diseases, University Clinical Center of Kosovo, Prishtinë, Kosovo; 2 University of Prishtina, Kongresi i Manastirit 3A, Prishtinë, Kosovo

**Keywords:** Tuberculous meningitis, Pregnancy, Intracerebral tuberculomas, Tuberculosis

## Abstract

**Background:**

Tuberculous meningitis is globally highly prevalent and is commoner in resource-limited countries and in patients with immunosuppression. Central nervous system tuberculosis is one of the severest forms of extrapulmonary tuberculosis during pregnancy and associated brain tuberculomas have been rarely reported. With the availability of neuroimaging at our hospital center, we present the first case of tuberculous meningoencephalitis associated with brain tuberculomas during pregnancy.

**Case presentation:**

In this case report we present a 25-year-old, Albanian, pregnant woman living in an urban area in Kosovo, who at 24 weeks of twin pregnancy manifested signs and symptoms of meningoencephalitis with decreased level of consciousness, hemiparesis, and generalized recurrent seizures. Based on medical history, origin from a region of high prevalence of tuberculosis, clinical presentation, especially neurological examination, cytobiochemical changes in cerebrospinal fluid (mild mononuclear pleocytosis with decreased level of glucose and elevated proteins), and elevated level of interferon-gamma release assay in cerebrospinal fluid, antituberculous therapy was initiated on the fourth day of admission. After 3 weeks of treatment, at 27 weeks of pregnancy, she had a preterm delivery and both twins, with low birthweight, died after 24 and 72 hours. Although findings on chest radiography were normal, brain magnetic resonance imaging showed signs of meningoencephalitis and multiple intracerebral tuberculomas, while Koch’s bacillus was isolated from urine cultures. On long-term follow-up after delivery, she was cured with no sequelae and became pregnant again without any additional complications.

**Conclusions:**

In countries with a high prevalence of tuberculosis, screening for central nervous system tuberculosis should be considered in the differential diagnosis of meningitis in pregnancy. Cerebral imaging is essential to establish the diagnosis of brain tuberculomas in such a case of suspected tuberculous meningoencephalitis during pregnancy.

## Background

Tuberculous meningitis (TBM), which accounts for approximately only 6% of all cases of extrapulmonary tuberculosis (TB), is one of the most serious clinical forms of TB, with a high mortality rate and disabling neurological sequelae [1, 2]. Diagnosis continues to be difficult particularly in resource-limited settings, and this may be truer in the setting of pregnancy [3].

Clinicians should also be aware of atypical presentation of TBM in pregnancy, and the suspicion of TBM may be sufficient grounds to initiate empirical antituberculous therapy [3].

TB of the central nervous system (CNS) includes TBM, spinal tuberculous arachnoiditis, and intracranial tuberculomas [4]. Intracerebral tuberculomas usually manifest as ring-enhancing of nodular lesions on magnetic resonance imaging (MRI) [5]. Intracranial tuberculomas are understood to be caused by hematogenous spread of bacillus into the brain, establishing tubercles that can coalesce and grow [6]. Tuberculomas are usually solitary lesions, but 15 to 34% are multiple [5].

TB is associated with a poorer outcome of pregnancy, although this may be due to the general risk factors for TB, namely poverty, malnutrition, and overcrowding [7]. Since pregnancy has not been shown to increase the risk of TB, the epidemiology of TB in pregnancy reflects the general incidence of disease [8]. The pathogenesis of TB infection and disease in pregnant women is similar to that in non-pregnant women [9, 10]. However, TB in pregnant women can present insidiously, since symptoms of malaise and fatigue may be attributed to pregnancy rather than disease [11]. The clinical signs of CNS TB are subacute and some of them might be confused with presentation of other diseases related with pregnancy such as pre-eclampsia, hyperemesis, brain tumors, and even epilepsy during gestation. TBM in Kosovo is known as a youth disease involving the young population. From a previous study of the 316 patients treated for TBM during a period of 8 years, 265 patients (84%) were younger than 30 years and there were five cases of TBM during pregnancy (1.6%) [12].

## Case presentation

### Patient information

A 25-year-old, Albanian, pregnant woman at 24 weeks of pregnancy, living in an urban area in Kosovo, was transferred from a Gynecology clinic because of vomiting, headache, decreased level of consciousness, and fever for last 2 weeks. She got pregnant with *in vitro* fertilization (IVF) after 6 years of marriage and during the pregnancy she had fatigue and vomiting frequently. She was treated earlier for urinary tract infection and salpingitis; a year ago she underwent surgery on her right fallopian tube (salpingostomy). She had no history of immunosuppression.

### Clinical findings

At admission, she was subfebrile, exhausted, hypotensive, adynamic, and anemic (height 165 cm, weight 65 kg). No palpable lymph nodes were noted; she had normal heart sounds, lungs with normal breath sound, blood pressure was 90/40 mmHg, respiratory rate 32 breaths per minute, pulse 96 beats/minute, and meningeal signs were positive. On neurological examination, deep tendon reflexes were hyperactive in her lower extremities, with patella and ankle clonus, and positive Babinski sign on both sides.

### Diagnostic focus and assessment

The differential diagnosis for intracerebral tuberculomas during pregnancy is not well established. A laboratory analysis showed moderate anemia (hemoglobin 11.0 g/dl), decreased level of total proteins (51 g/L), C-reactive protein (CRP) level reaching 52 mg/L, procalcitonin (PCT) level of 0.79, and erythrocyte sedimentation rate amounting to 95 mm/hour.

A TORCH (Toxoplasmosis, Other Agents, Rubella, Cytomegalovirus, and Herpes Simplex Virus)-panel test had negative results with negative immunoglobulins M and G for toxoplasmosis, cytomegalovirus (CMV), Epstein–Barr virus (EBV), rubella virus (RV), and herpes simplex virus 2 (HSV2). Human immunodeficiency virus (HIV) 1 and 2 antibodies were also negative, and *Brucella* and hepatitis serology testing were negative.

Blood cultures were sterile while urine cultures were positive (*Klebsiella* species). Cerebrospinal fluid (CSF) analysis showed mild mononuclear pleocytosis of 42 cells/mm^3^ (mononuclear cells, 100%); glycorrhachia, 1.7 mmol/L; proteinorachia, 2.0 mmol/L; and CSF/blood glucose ratio, 0.44. A repeated lumbar puncture (LP) after 48 hours gave clear CSF, 165 cells/mm^3^ (mononuclear cells 90%); glycorrhachia, 1.56 mmol/L; proteinorachia, 0.84 mmol/L; CSF/blood glucose ratio, 0.46 mmol/L; tuberculin skin test (TST) was negative; and microscopic examination for acid-fast bacilli of CSF and sputum revealed negative result.

### Therapeutic focus and assessment

She was treated the first 4 days with ceftriaxone and anti-edematous treatment (mannitol and dexamethasone). Due to a worsening of her clinical presentation with signs and symptoms of meningoencephalitis, duration of illness >2 weeks, medical history, origin from a region of high prevalence of TB, CSF criteria, and high level of interferon-gamma release assay (IGRA; 197.7 pg/ml; sent abroad) in CSF, treatment with four antituberculous agents was initiated: rifampicin (R), isoniazid (H), pyrazinamide (Z), and ethambutol (E). During the first 3 weeks of treatment she had decreased level of consciousness from somnolence to soporous state, and she manifested right hemiparesis and generalized recurrent seizures; an electroencephalogram (EEG) showed pathological findings.

**Fig. 1:**
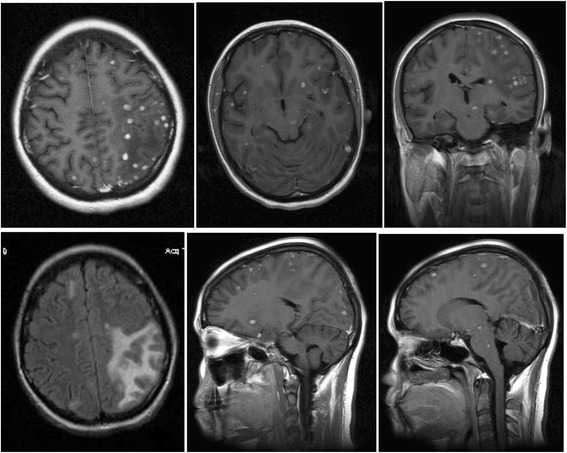
First magnetic resonance imaging brain images showing multiple intracerebral tuberculomas

After 3 weeks of treatment, at 27 weeks of pregnancy, she got vaginal bleeding and uterine contractions and after consulting gynecologist, underwent cesarean section. The preterm underweighted twins died: female baby 700 grams with Apgar score 2 died within 24 hours and male baby 800 grams with Apgar score 4 who was transferred outside the country died within 72 hours. We did not get any results of performed autopsy. The mother was then transferred back to the Infectious Diseases Clinic in Prishtina to continue the treatment. Postnatally, chest radiography was done which revealed no pathological findings while an ultrasound of her abdomen was normal. A MRI of her brain, realized 4 weeks after admission at the private clinic, showed multiple hyperintense nodular lesions suspected for intracerebral tuberculomas, brain edema in parieto-temporo-occipital region on left side, augmented subarachnoid space, and ischemia at left parietal lobe suggesting meningoencephalitis. Her cortical-subcortical fronto-temporo-parietal regions were involved bilaterally, and there was a white mass with predominance in parieto-temporo-occipital regions on left side, bilateral cerebellar hemispheres, pons, mesencephalon, and dilatation of subarachnoidal space (Fig. 1).

**Fig. 2:**
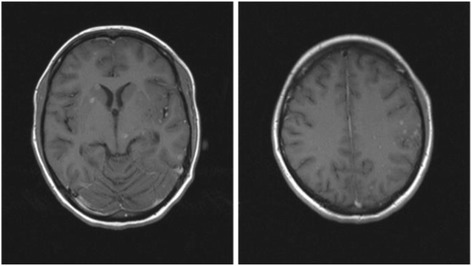
Second magnetic resonance imaging brain images showing regression of the size and number of intracerebral tuberculomas

After 6 weeks, cultures of CSF and sputum on Lowenstein were negative, whereas cultures of urine on Lowenstein were positive three times and a sensitivity test showed sensitivity to all tested antituberculous agents: R, H, E, Z, and streptomycin (S). Elevated pleocytosis in CSF lasted for 53 days, hypoglycorrhachia for 26 days, and elevated proteins for 70 days. A repeated MRI of her brain after 3 months showed regression of the size and number of tuberculomas (Fig. 2).

She was treated for 12 months with antituberculous agents with no sequelae. After 2 years she got pregnant again by IVF and has twin girls.

## Discussion

TBM continues to threaten inhabitants of Kosovo as a developing country with poor socio-economic standards. A previous study showed that a decade before the 1999 war in Kosovo there were approximately 40 cases of TBM per year treated at the Infectious Diseases Clinic in Prishtina [12]. Of the total 316 patients treated for TBM during a period of 8 years, there were five cases of TBM during pregnancy (1.6%).

In the first decade after the war in Kosovo, the incidence of TBM decreased by 50% with a further decrease in the last few years. As noted before, young people less than 18 years of age are the age group most affected by TBM (64% in 2014). Kosovo is ranked among European countries with a high prevalence of TB. Based on published data, over the last few years there have been approximately 900 cases annually. With the availability of MRI in the last few years, brain tuberculomas are more frequently seen in patients with TBM, but its occurrence during pregnancy was not documented earlier at our clinical center.

The incidence of TB in pregnancy ranges between 1 and 2% [13]. The diagnosis and management of tuberculoma is an important public health problem in both developing and industrialized nations but unlike pulmonary TB, which has been under close investigation, the diagnosis and treatment of tuberculomas have received little consideration [14]. Intracranial tuberculomas are the least common presentation of CNS TB, found in 1% of these patients [15]. Multiple CNS tuberculomas in an immunocompetent patient may closely resemble metastatic malignancy [16].

Our patient was HIV negative and had no history or indication of immunosuppression. Tuberculomas usually represent reactivation of a latent tuberculous focus; sometimes after many years [17]. Our patient was treated for primary sterility and underwent surgery on her right fallopian tube (salpingostomy). During the pregnancy after IVF, at 24 weeks of pregnancy she developed sign and symptoms of TB meningoencephalitis, without a TB pulmonary focus. Pregnancy itself appears to be a risk factor for developing TB [18]. The increased susceptibility to TB may be due to immunological changes in pregnancy. Pregnancy partially suppresses T helper type 1 (Th1) cell-mediated immunity in favor of the antibody response, which is T helper type 2 (Th2) mediated, perhaps to protect the fetus from immunological rejection [18]. Cell-mediated immunity has a dominant role in protection against *Mycobacterium tuberculosis* and active TB is associated with a dominant Th2 immune response [18].

The treatment with antituberculous agents (R, H, E, and Z) was started during the first week based on anamnesis (duration of illness was 2 weeks, history of treatment for salpingitis and sterility, originated from a region with a high prevalence of TB), clinical criteria with neurological features, CSF criteria (mild mononuclear pleocytosis, proteins >1 g/L, and CSF to blood glucose ratio of less than 0.5), and a high level of IGRA in CSF. Studies in adults and children using IGRA have shown this assay to have a specificity of 98% or greater and sensitivity that is relatively comparable to TST, approximately 80% [19–22]. Validating IGRA during pregnancy is important because an altered immune response occurs during pregnancy and IGRA is dependent on an individual’s Th1 response to *M. tuberculosis* antigens [23–25].

The benefits of treating active TB in pregnancy dramatically outweigh any potential drug toxicity [26]. IGRA, QuantiFERON-TB Gold (QFT-G), polymerase chain reaction (PCR), and other rapid TB diagnostic tests could not be done at our clinical center and late cultures continue to influence and delay the treatment and the outcome of patients with TBM. As reported before, clinicians should also be aware of atypical presentation of TBM in pregnancy, and the suspicion of TBM may be sufficient grounds to initiate empirical antituberculous therapy [3]. CSF and sputum cultures revealed negative results after 6 weeks, while *M. tuberculosis* was identified in urine cultures after 6 weeks. *M. tuberculosis* is reportedly identified by staining for acid-fast bacilli in a CSF smear in only 10 to 20% of patients with TBM, whereas the pathogen is found by mycobacterial culture in 25 to 80% of patients with TBM [1]. From our previous studies, a mycobacterium culture resulted positive only in 10% of cases [12].

During the bacillemia that follows primary infection or late reactivation TB, the chance occurrence of a subependymal tubercle, with progression and rupture into the subarachnoid space, is the critical event in the development of TBM [27]. After 3 weeks of treatment with antituberculous agents, she required emergency caesarean section. Her chest radiography showed normal findings whereas MRI images realized after 4 weeks of treatment for TBM, showed signs of meningoencephalitis and multifocal distribution of tuberculomas. Tuberculomas can exhibit as a single large mass or as multiple masses throughout the brain, and are more likely to be found in the posterior fossa [28]. In this case, whole brain regions were involved with predominance on the left side. In multiple cerebral space-occupying lesions, a differential diagnosis should include malignant lesions, pyogenic abscesses, sarcoidosis, toxoplasmosis, and cysticercosis. From our experience at our clinical center, we have rarely seen development of obstructive hydrocephalus during TBM in adults. In the last 15 years, only one patient, 21 years of age, manifested obstructive hydrocephalus. By contrast, obstructive hydrocephalus was the most frequent neurological complication of TBM in children registered in 14% of cases [12].

There are only a few case reports of intracerebral tuberculomas during pregnancy (Table 1) [13–15, 29–31]. Patients who presented intracerebral tuberculoma during pregnancy were younger than 30 years of age and 57% of them had multiple intracerebral lesions. As reported before from our previous study during an 8-year period, 84% of patients treated for TBM were younger than 30 years of age [12].

**Table 1:** Reported cases of cerebral tuberculomas during pregnancy

Case reports	Patient’s age	Gestational age of pregnancy	The outcome of pregnancy	Number of cerebral tuberculomas
Feenstra *et al*. (1999) [30]	28 years	Third trimester	Caesarean section	Multiple lesions
Pandole *et al*. (2001) [31]	19 years	Third trimester	Caesarean section	Multiple lesions
Gasparetto *et al*. (2003) [29]	17 years	Third trimester	Normal delivery	Multiple lesions
Ahmadi *et al*. (2011) [14]	25 years	Third trimester	Normal delivery	Solitary lesion
Muin *et al*. (2015) [15]	19 years	Second trimester	Normal delivery	Solitary lesion
Arumugam *et al*. (2016) [13]	24 years	Third trimester	Normal delivery	Solitary lesion
Namani *et al*. Presented case	25 years	Second trimester	Caesarean section	Multiple lesions

Five cases (71%) presented the disease during the third trimester of pregnancy, while two cases presented the disease during the second trimester, including our patient. Most cases (71%), including our patient, manifested the disease during their first pregnancy. Four patients (57%) had normal delivery while three patients underwent urgent caesarean section, including our patient. All neonates survived, except the underweight twins of our patient with low Apgar scores (700 g and 800 g).

Tuberculomas often present with symptoms and signs of focal neurological deficit without evidence of systemic disease [32]. They behave like space-occupying lesions and present with signs of raised intracranial pressure, localized neurological deficits, seizures, and even behavioral problems [13]. Seizures are one of the most common symptoms occurring in up to 85% of cases along with symptoms related to elevated intracranial pressure such as headache, papilledema, and lethargy [33]. Our patient presented signs and symptoms of meningoencephalitis and space-occupying lesions: headache, vomiting, fever, altered mental status to soporous state, right hemiparesis, and generalized seizures. A total of 71% of reported cases of intracerebral tuberculomas manifested neurologic deficit and seizures (Table 1) [13–15, 29–31]. CSF analysis showed mild mononuclear pleocytosis as in only one of the previously reported cases (28.6%) [14]. The presented case (Namani et al.) and one previosly reported case are equal to 2. 2/7 cases=28.6%. Four cases in Table 1 (57%) used aggressive methods for confirming the diagnosis of intracerebral tuberculomas during pregnancy: in two cases, brain biopsy [29, 30]; and in two cases, surgical intervention [14, 31].

Reactivation of latent TB during pregnancy must be taken into consideration. The reactivation of latent TB in our case occurred from urinary tract TB with positive urine cultures for *M. tuberculosis.* She underwent surgery on her right fallopian tube a year ago and was treated with quinolones for urinary infection. During pregnancy, TB is associated with poor outcomes, including increased mortality in both the neonate and the pregnant woman [34]. TB that is confined to the thorax or limited to lymphadenitis poses a minor risk to the fetus, while adverse fetal outcomes are more frequent with extrapulmonary disease [35]. During pregnancy, vertical transmission from an affected mother to her fetus is extremely rare. However, there is a sixfold increase in perinatal death and a twofold risk of premature birth and low birthweight [36]. Pregnant women with extrapulmonary TB (excluding lymphadenitis) have been found to have significantly higher frequencies of low birthweight babies and babies with Apgar scores <7 [28]. Toxemia (pre-eclampsia), vaginal bleeding, fetal death at 16 to 28 weeks, acute fetal distress, prematurity (<37 weeks), small for date, low birthweight (<2.5 kg), and perinatal death have all been described, but are also associated with poverty, malnutrition, and overcrowding factors that are themselves associated with TB [18, 37]. Both twins of our patient had a low birthweight and Apgar scores. S should not be used in pregnancy because of possible ototoxicity in the fetus, while Z is generally not recommended because of a lack of data on the risk for teratogenicity [26]. Based on her severe clinical presentation we used four antituberculous agents including Z (R, H, E, and Z) and dexamethasone during first 6 weeks. After 3 months of treatment, there was still evidence of intracerebral tuberculomas although MRI showed that they were fewer and smaller compared to the first MRI images. It is universally accepted that anti-TB drugs are essential for the successful treatment of intracranial tuberculomas but there is no agreement regarding the duration of therapy [36, 38, 39]. The United States (US) Centers for Disease Control and Prevention recommends 12 months of treatment for CNS TB when the *M. tuberculosis* strain is sensitive to all drugs [40]. Total resolution of the tuberculomas is observed when scans demonstrate no enhancing lesions or only an area of calcification [38].

Sputum with CSF microscopic examination for acid-fast bacilli, negative TST, and chest X-ray did not help much, whereas clinical presentation, cytobiochemical changes in CSF, IGRA, and brain MRI were crucial for diagnosing TBM during pregnancy.

## Conclusions

In countries with a high prevalence of TB, screening for CNS TB should be considered in the differential diagnosis of meningitis in pregnancy. Cerebral imaging is essential to establish the diagnosis of brain tuberculomas in such a case of suspected tuberculous meningoencephalitis during pregnancy.

## Abbreviations

CMV: Cytomegalovirus
CNS: Central nervous system
CRP: C-reactive protein
CSF: Cerebrospinal fluid
E: Ethambutol
EBV: Epstein–Barr virus
EEG: Electroencephalogram
H: Isoniazid
HIV: Human immunodeficiency virus
HSV2: Herpes simplex virus 2
IGRA: Interferon-gamma release assay
IVF: *In vitro* fertilization
LP: Lumbar puncture
MRI: Magnetic resonance imaging
PCR: Polymerase chain reaction
PCT: Procalcitonin
QFT-G: QuantiFERON-TB Gold
R: Rifampicin
RV: Rubella virus
S: Streptomycin
TB: Tuberculosis
TBM: Tuberculous meningitis
Th1: T helper type 1
Th2: T helper type 2
TST: Tuberculin skin test
Z: Pyrazinamide
